# Pediatric Intracranial Abscess From Sinusitis Leading to Status Epilepticus: A Clinical Case Report

**DOI:** 10.7759/cureus.52116

**Published:** 2024-01-11

**Authors:** Brian Bartlett, Luke Crance, Jennifer Myaeng, Ashley Anderson

**Affiliations:** 1 Emergency Medicine, Mayo Clinic, Mankato, USA; 2 Pediatrics, Mayo Clinic, Mankato, USA

**Keywords:** focal neurological deficits, altered mental status, status epilepticus, sinusitis, pediatric intracranial abscess

## Abstract

This case study presents the rare incidence of an eight-year-old female with a pediatric intracranial abscess, a life-threatening complication of sinusitis. The patient manifested focal neurological deficits and experienced status epilepticus before presenting to the Emergency Department. She had been diagnosed with sinusitis and prescribed amoxicillin one day prior. The case underscores the importance of early recognition and intervention in managing this rare but potentially fatal complication of a common pediatric illness.

## Introduction

This case report describes an uncommon complication of a common pediatric illness. Sinusitis is a common occurrence in the pediatric population and can occasionally lead to severe complications, including intracranial abscesses [1]. These abscesses are infections within the brain parenchyma, often resulting from contiguous spread of infection from the paranasal sinuses [2].

Pediatric intracranial abscesses are associated with high rates of morbidity and mortality, making timely diagnosis and management crucial [3]. Neurological findings are central to the diagnosis, with symptoms such as headache, fever, and altered mental status often present. In the pre-antibiotic era, approximately 32% of intracranial abscesses were secondary to a sinogenic source. A study by Tandon et al. compared the incidence of intracranial abscesses secondary to sinusitis in patients from 1950 to 1979 (after the discovery of antibiotics) and from 1980 to 2004. The incidence was 15.7% and 14.6%, respectively [4]. 

The incidence of seizures in pediatric patients with intracranial abscesses from sinusitis varies significantly across studies, ranging from 15% to 62% [5]. This variability may be due to differences in patient selection, clinical presentation, and diagnostic criteria across studies [6].

## Case presentation

An eight-year-old, otherwise healthy and fully vaccinated, female patient from rural Minnesota presented to local emergency department after emergency medical services were called for possible seizure and altered mental status. In the preceding five days, the patient developed a significant headache and a fever of 39.4°C. The patient’s mother had recently been diagnosed with streptococcal pharyngitis and even though the patient did not have a positive test, she was prescribed amoxicillin by an outside facility two days prior to emergency department arrival. The parents also reported that the patient developed a left facial and eyelid droop with associated facial swelling that morning. Later that day the patient was less responsive according to the mother and developed shrugging movements of the shoulders leading to tonic-clonic motor activity. Due to this, emergency medical services were called and upon arrival found the patient to be in respiratory distress and hypoxemic with an oxygen saturation of 85%. A non-rebreather face mask was placed at 15 liters per minute and the patient was transported to the emergency department.

Upon initial presentation the patient was unresponsive, tachypneic, and had shallow breathing. The vital signs included: oxygen saturation level of 91% on 15 liters facemask, sinus tachycardia with a rate of 141, skin temperature of 37.4°C, and a respiratory rate of 36. She exhibited tonic-clonic movements on arrival and was intubated with propofol and rocuronium for airway protection. Post-intubation, a propofol infusion was administered for sedation and seizure control. Initial labs were notable for leukocytosis of 11.9 and lactate of 3.2 as well as transaminitis (Table 1).

**Table 1:** Pertinent initial laboratory findings

Lab	Normal Range	Value
Aspartate Aminotransferase (AST)	8-50 (U/L)	587
Alkaline Phosphatase	142-335 (U/L)	529
Alanine Aminotransferase (ALT)	7-45 (U/L)	426
Bilirubin, Total	<= 1.8 mg/dL	1.8
Leukocytes	3.8 - 10.4 x10(9)/L	11.9
Lactate	<=2.2 mmol/L	3.2

**Figure 1:**
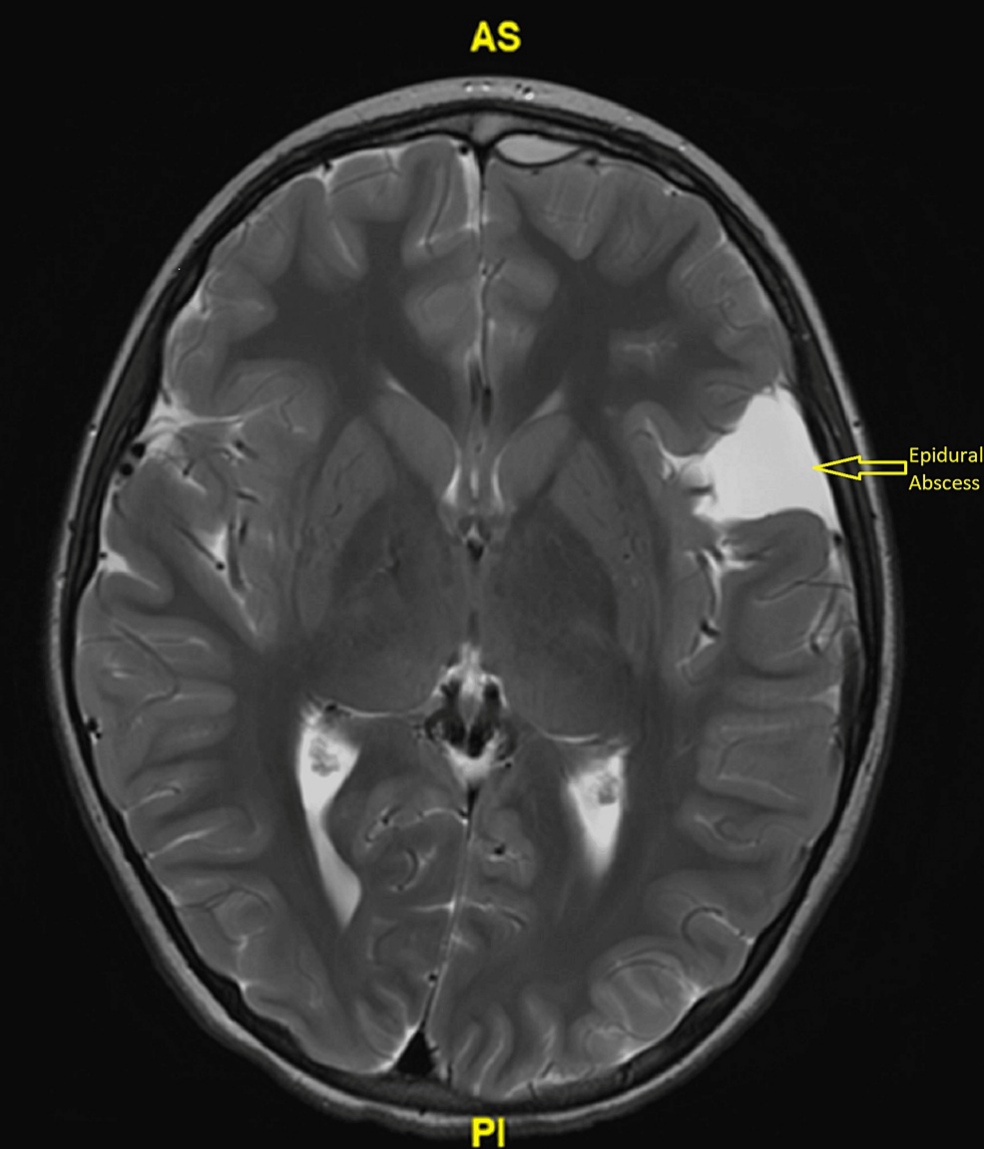
Axial view of brain MRI demonstration small left frontal epidural abscess

Non-contrast computed tomography (CT) of the head, as well as CT angiography of the head and neck, were obtained showing pansinusitis and arachnoid cyst. Lumbar puncture was also obtained and was unremarkable for an infectious source showing only one total nucleated cell, glucose of 71, and protein of 18. She was administered 50 mg/kg of IV ceftriaxone, 15 mg/kg of vancomycin, and 15 mg/kg of IV acyclovir and transferred to the Pediatric Intensive Care Unit. She was weaned from the ventilator and extubated. Further antibiotic coverage was added with metronidazole and a levetiracetam loading dose of 50 mg/kg was also administered as her initial presentation was concerning for seizure. Upon admission, Magnetic Resonance Imaging (MRI) of the brain was obtained showing complicated sinusitis resulting in small left front epidural abscess signal enhancement concerning for meningitis and left frontal bone enhancement and edema concerning for osteomyelitis (Figure 1). The Neurosurgical team was consulted but no neurosurgical intervention was required. 

A throat swab done upon admission was positive for Group A* Streptococcus*. On day two of hospitalization, she underwent sinus debridement and cultures were obtained which also grew *Streptococcus pyogenes*. She improved and was able to be discharged on day six of hospitalization with a peripheral central catheter line for continuation of ceftriaxone (2 grams daily). Oral metronidazole (250 mg three times daily) and levetiracetam (400 mg twice daily) were continued. 

**Figure 2:**
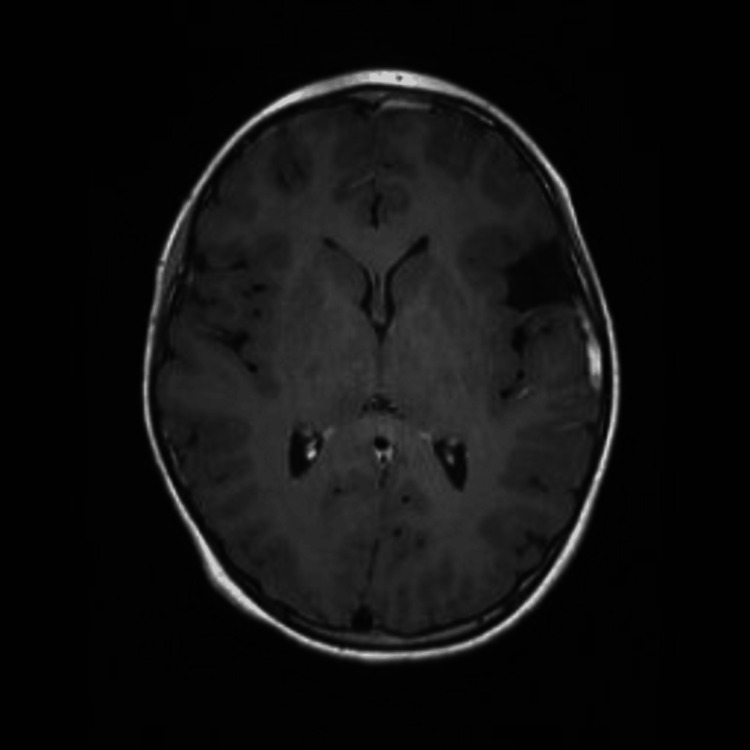
Axial, non-contrast MRI three weeks after hospital admission reveals resolving epidural abscess.

Post-hospitalization follow-up with an infectious disease specialist occurred six days after discharge and she was transitioned to oral levofloxacin (250 mg daily) and continued the metronidazole (250 mg three times a day) to complete a total of six weeks of antimicrobial therapy. Her peripheral inserted central catheter was removed. She continued to demonstrate clinical improvement and repeat outpatient MRI without contrast showed resolving epidural abscess (Figure 2).

Three months after hospital discharge, she had a normal electroencephalogram and levetiracetam was discontinued. This patient had an excellent clinical outcome and was able to return to her baseline with normal functioning.

## Discussion

This case report brings into focus an uncommon pediatric complication of sinusitis: epidural abscess caused by *Streptococcus pyogenes*. Acute sinusitis is a common illness in the pediatric population and is easily treatable. Complications from this illness are rare, but when present are typically infections involving the orbital and intracranial spaces [6]. Orbital complications, the most common, account for 60-75% of complicated sinusitis. The most common intracranial infections are subdural empyema, epidural abscess, cerebral abscess, and meningitis. Other intracranial complications include cerebritis, superior sagittal or cavernous sinus thrombosis [7,8].

The predominant organisms responsible for uncomplicated acute sinusitis in children are non-typeable *Haemophiles influenzae, Streptococcus pneumoniae, *and* Moraxella catarrhalis* [8]. These are also the bacteria that commonly cause acute otitis media in pediatric patients. In complicated sinusitis, the primary pathogen is often different. *Streptococcus anginosus *group bacteria were the primary pathogens, accounting for 34% of cases, in a retrospective case series examining pediatric sinusitis with intracranial complications. This study was conducted at a single institution from 2007 to 2019. In contrast, beta-hemolytic streptococci constituted only 2% of the cases[9]. In a single-center case series from 2006 to 2016 focusing on complicated pediatric sinusitis requiring surgical intervention, the infections were predominantly polymicrobial. The most common pathogens identified were *Streptococcus, Staphylococcus*, and anaerobes [10].

In this clinical scenario, pivotal considerations included the execution of a lumbar puncture and the potential use of intravenous steroids following the diagnosis of a brain abscess. Although dexamethasone is often used for cerebral edema from brain abscess, its impact on mortality remains uncertain. It wasn't considered during the initial Emergency Department assessment. A meta-analysis combining seven cohorts and four case series found no significant mortality difference in brain abscess patients treated with dexamethasone versus standard care without steroids [11]. The patient's symptoms, including fever, headache, seizure-like activity, and recent sinusitis, warranted a broad differential diagnosis, including meningitis. The CT scan showed a left-sided arachnoid cyst but no midline shift. After discussion with the Pediatric Intensive Care Physician, lumbar puncture was conducted as meningitis remained on the differential and there was no evidence of midline shift. However, a post-admission MRI with contrast identified the abscess, correcting the initial diagnosis of arachnoid cyst. In patients presenting with altered mental status, papilledema, age over 60, immunocompromised status, high suspicion for subarachnoid hemorrhage, recent seizure, or new focal neurological deficit, careful imaging selection is advised [12].

## Conclusions

Intracranial complications of acute sinusitis are rare but must be considered when a pediatric patient presents with new neurologic symptoms in the setting of recent sinusitis. The complexity of the eight-year-old patient's presentation, characterized by seizures, altered mental status, and focal neurological deficits, offers a unique perspective on the potential consequences of a common pediatric illness. Timely diagnosis, prompt initiation of antibiotic therapy, and surgical intervention were instrumental in averting a poor outcome, as intracranial abscesses carry a significant risk of mortality and neurological morbidity.

The report also underlines the importance of the pathogen in complicated sinusitis. This intracranial epidural abscess was caused by Streptococcus pyogenes, highlighting that bacterial pathogens causing uncomplicated sinusitis may differ from those implicated in complicated cases. In conclusion, this case serves as a strong reminder to clinicians to maintain a high degree of clinical suspicion and respond quickly to evolving symptoms in pediatric sinusitis. Continued research and collection of case reports such as this one will continue to enhance our understanding and management of rare but serious complications of common pediatric illnesses.
